# Patient Safety in Surgery: scaling the journal’s global visibility and scientific renown

**DOI:** 10.1186/s13037-024-00416-w

**Published:** 2024-12-09

**Authors:** Philip F. Stahel

**Affiliations:** 1 Mission Health, 50 Schenck Pkwy, Asheville, NC 28803 USA; 2 Department of Surgery, East Carolina University Brody School of Medicine, Greenville, NC 27834 USA; 3 Department of Specialty Medicine, Rocky Vista University College of Osteopathic Medicine , Parker, CO USA; 4 East Carolina University Brody School of Medicine, 600 Moye Blvd, Greenville, NC 27834 USA

**Keywords:** Patient safety in surgery, Publication metrics, Peer review process, Journal citation reports

Since the journal’s founding in 2007, *Patient Safety in Surgery* (Fig. 1) has seen an impressive incremental improvement in publications metrics and scientific reputation [1]. At present, the journal continues to sustain a global readership, with the exception of selected countries in Central/West Africa and Greenland (Fig. 2). This success is largely owed to the open-access paradigm which continues to grow in popularity as an alternative publication platform to standard print journals in the 21st century [2]. Online open-access publications provide several intuitive advantages to authors and readers. In essence, the open-access modality provides the timely and unrestricted access to scientific knowledge to a global audience in absence of journal subscription charges or copyright release requirements [2]. All open-access articles are free to download, copy, reproduce and distribute per the Creative Commons (CC) license, with the only stipulation to cite the original source of publication [3]. From a submitting author’s perspective, there are multiple compelling arguments to consider open-access publishing. First, there is no page limit to the article length and number of tables and figures included, and there are no extra charges for publishing color images or embedded video links. More importantly, the authors retain the full copyright of their published articles, which allows replicating data and figures without the need to request a formal copyright release by the publisher [3]. In response to changing author needs and the increasing risk of artificial intelligence (AI) tools being utilized for manipulation of data and images, all Springer-Nature journals (including *Patient Safety in Surgery*) have moved to an alternative CC license by default, termed CC-BY-NC-ND. This publication license ensures that authors have greater control over the reproduction and distribution of the content in their respective articles. Authors retain the liberty to select the traditional CC-BY license by request, should this remain their individual licensure preference.

**Fig. 1:**
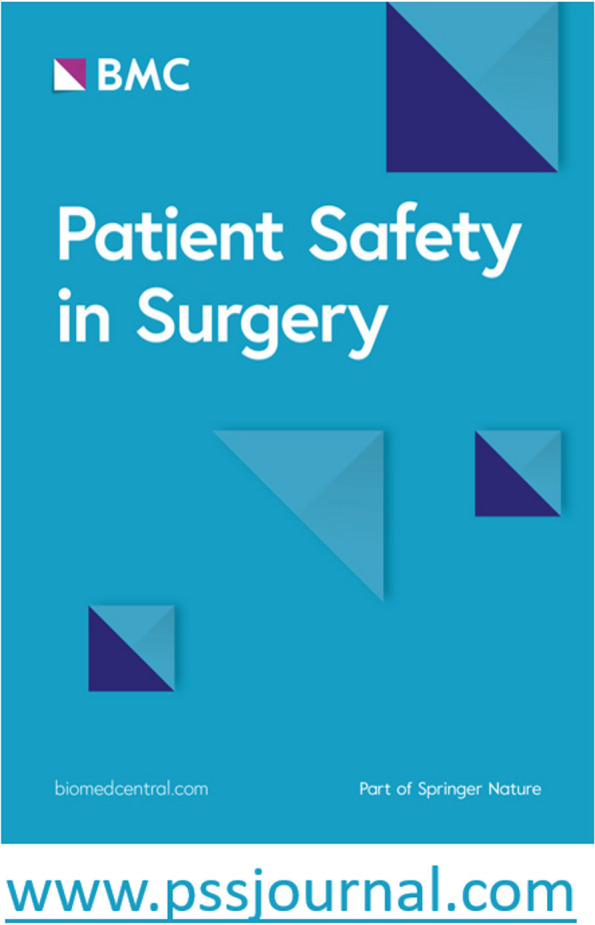
Patient Safety in Surgery is a peer reviewed open-access journal published by BMC/Springer Nature since 2007

**Fig. 2:**
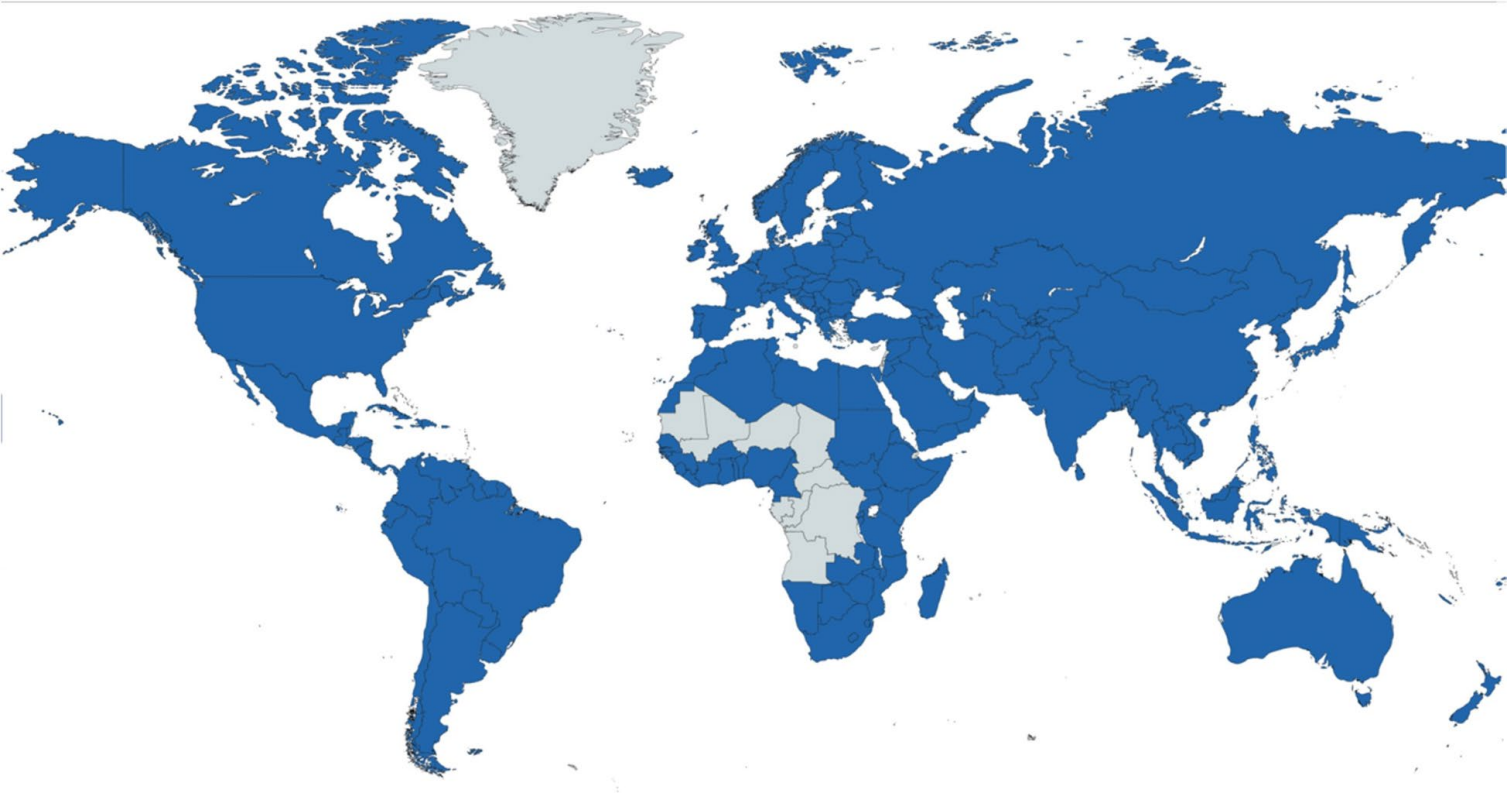
The journal’s global readership in 2023. (Source: BMC/Springer Nature)

Finally, the fast-track submission to publication process enables quicker turn-around times of submitted manuscripts and timely dissemination of scientific work (Fig. 3**)**. *Patient Safety in Surgery* prides itself in providing authors with detailed feedback on their submissions, complying with COPE guidelines to ensure complete and proper peer review for all submissions in a timely manner. The current rejection rate for all submitted manuscripts is around 70% (2023). This relatively high rate reflects on the journal’s rigorous peer review process and high publication acceptance standards. The journal furthermore provides specific guidance with practical tips and tactics for submitting authors aimed at addressing frequent shortcomings and improving the scientific content and the overall quality of submitted manuscripts [4].

**Fig. 3:**
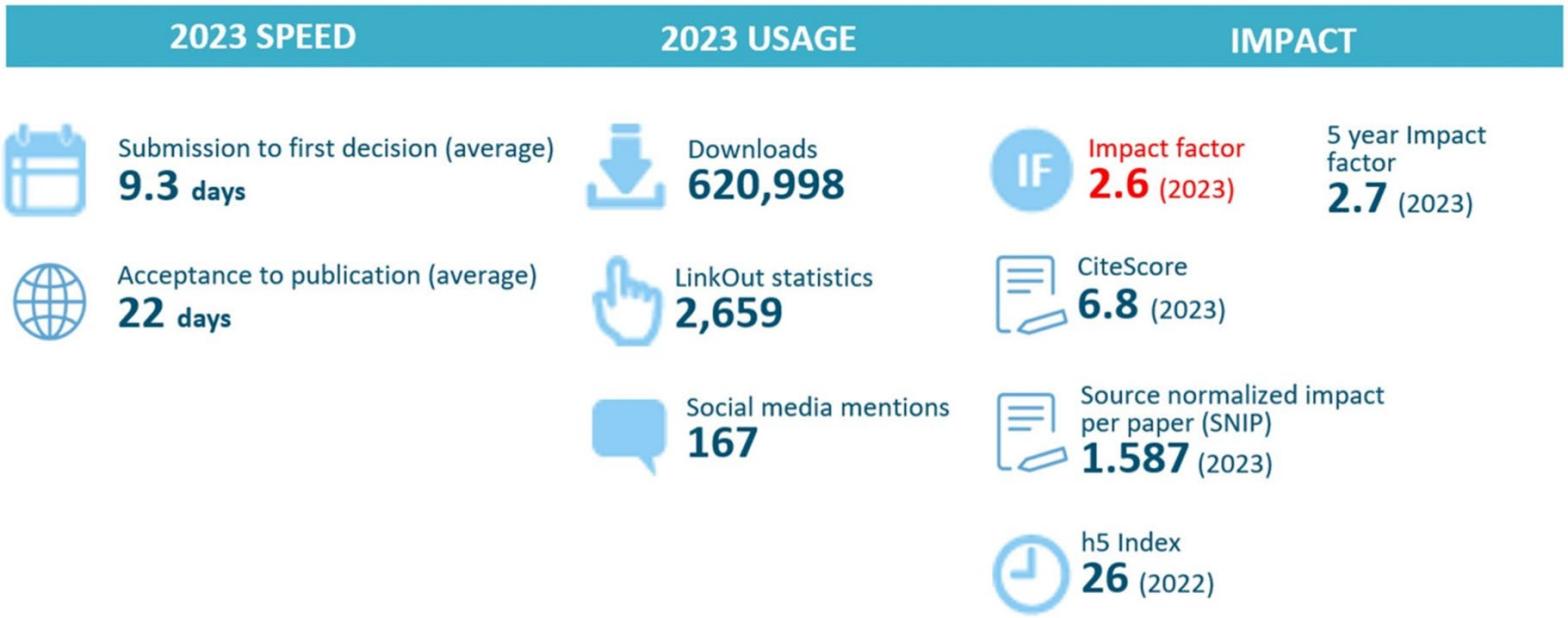
Patient Safety in Surgery publication metrics for 2023. (Sources: BMC/Springer Nature; Journal Citation Reports™ Clarivate 2023)

The Editorial Board of *Patient Safety in Surgery* is geographically diverse and internationally recognized based on each editor’s respective field of expertise [5]. In addition, the nationality of authors who publish in the journal comprises more than 50 different countries from around 300 different institutions, until present. The variety of authors’ countries of origin for articles published in the journal during the calendar year 2023 is depicted in Fig. 4. The journal’s official impact factor of 2.6 (2023) by Clarivate Journal Citation Reports™ further substantiates the scientific credibility as it pertains to the journal’s mission and renown. Notably, at the time of drafting this editorial, the highest impact article published in the journal had 257,000 accesses and 260 citations since its original publication date in 2010 [6]. In addition, the fastest cited article published during the novel coronavirus pandemic in 2021 had 226,000 accesses, 134 citations, and 355 Altmetric mentions, until present [7].

**Fig. 4:**
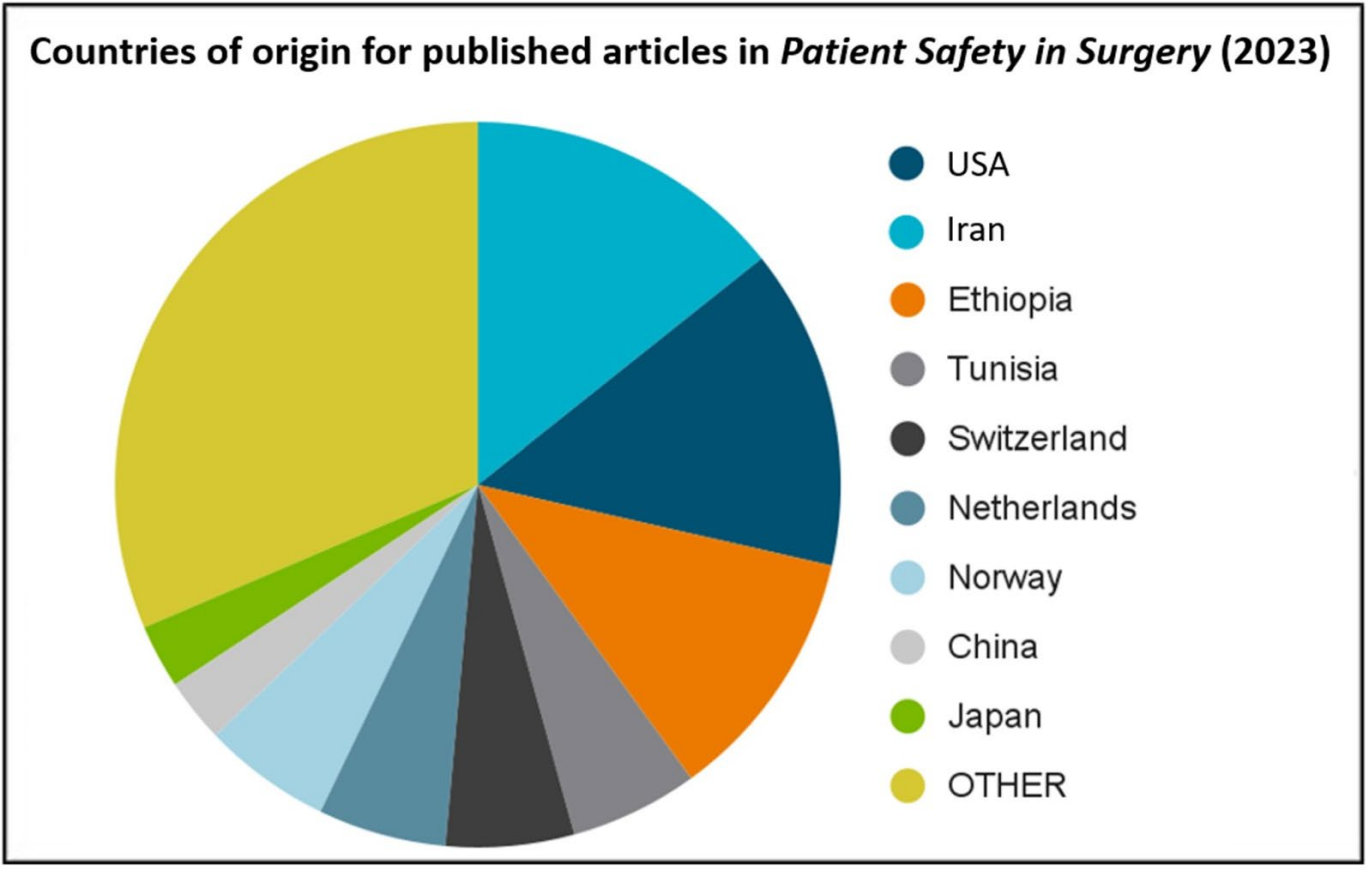
Top-10 countries of origin for published articles in 2023. (Source: BMC/Springer Nature)

*Patient Safety in Surgery* recently started expanding the publication portfolio by commissioning guest editors for theme-based special article collections. Our first article collection is dedicated to “Machine learning approach for improvement of patient safety in surgery” [8] as a tribute to the expanding role of artificial intelligence and predictive analytics for patient safety. In addition, we are currently partnering with our sister journal *World Journal of Emergency Surgery* [9] to promote a special issue on “The global impact of antibiotic resistance on emergency surgery and patient safety” in partnership between the two BMC/Springer Nature journals [10]. The expanding scale and scope of *Patient Safety in Surgery*, in conjunction with a robust peer review process, fast article turnaround times, and respectable impact factor (2.6) will continue to support the journal’s appeal as a publication target of choice for new authors and our global readers.

## Data Availability

Please contact the author for data requests.
